# Surgical management of renal cell carcinoma with extension into the inferior vena cava and right atrium: A case report

**DOI:** 10.1016/j.ijscr.2025.112117

**Published:** 2025-10-26

**Authors:** Muhammad Arza Putra, Agus Rizal Ardy Hariandy Hamid, Fakhri Ramhan, David Hutagaol, Konda Kinanti Muroso, Renaldi Prasetio

**Affiliations:** a Division of Thoracic, Cardiac and Vascular Surgery, Department of Surgery, Faculty of Medicine, University of Indonesia, Cipto Mangunkusumo Hospital, Jakarta, Indonesia; b Department of Urology, Faculty of Medicine, University of Indonesia, Cipto Mangunkusumo Hospital, Jakarta, Indonesia; c Resident of Thoracic, Cardio and Vascular Surgery, Faculty of Medicine, University of Indonesia, Cipto Mangunkusumo Hospital, Jakarta, Indonesia

**Keywords:** Renal cell carcinoma, Tumor thrombus, Right atrium, Cardiopulmonary bypass, Deep hypothermic circulatory arrest, Surgical management, Case report

## Abstract

**Introduction:**

Metastatic heart tumors are more prevalent than primary cardiac tumors. Renal cell carcinoma (RCC) is one of the tumor that can spread to the Inferior Vena Cava (IVC) to the right heart chamber. We present the Case of RCC with extension into the IVC and Right Atrium with surgical treatment and approach.

**Case description:**

40-years old male experienced shortness of breath 3 months prior. PET CT-scan showed Lobulated hypermetabolic mass with malignant aspect measuring ±68.6 × 64.9 mm with necrotic area in the right renal lower pole corresponds to RCC. Tumor thrombus (TT) of the inferior vena cava reaches the right atrium. Transthoracic echocardiography showed multiple lobe thrombus from the IVC entering the RA. Surgical therapy with excision of right kidney tumor and TT into the IVC and RA with cardiopulmonary bypass (CPB).

**Clinical decision:**

The thrombus was Mayo grade 4, preoperative imaging and intraoperative findings confirmed a short, mobile, non-adherent thrombus without caval wall invasion. Complete removal was achieved with CPB alone, omitting DHCA. Intraoperative transoesophageal echocardiography guided safe extraction, reducing procedural complexity and avoiding DHCA-related risks.

**Conclusion:**

Management of RCC with RA extension requires precise assessment of thrombus level, morphology, and metastatic status. In selected Mayo grade 4 cases without wall adhesion, friable thrombus, or valve involvement, CPB without DHCA is a safe alternative, minimizing perioperative morbidity. DHCA should be prioritized when adhesion, friability, or valve extension is present.

## Highlights

• Metastatic heart tumors are more prevalent than primary cardiac tumors
• Renal cell carcinoma (RCC) is one of the tumor that can spread to the Inferior Vena Cava (IVC) to the right heart chamber.
• Surgical management of RCC with tumor thrombus (TT) can be performed under cardiopulmonary bypass (CPB), with or without deep hypothermic circulatory arrest (DHCA), depending on thrombus morphology and wall invasion.
• Selected Mayo grade 4 cases without caval wall adhesion or thrombus fixation may be safely managed with CPB alone, while DHCA should be reserved for complex or adherent thrombi.

## 1 Introduction

Metastatic heart tumors are more prevalent than primary cardiac tumors. The incidence of primary heart tumors is 0.14 %, while that of metastatic heart tumors is 6 %. One type of metastatic heart tumor that can spread from the renal vein into the inferior vena cava (IVC) and ultimately into the right ventricular chambers is renal cell carcinoma (RCC) [1,2]. RCC is the most common kidney cancer which is more common in men. 4–10 % of patients with renal neoplasms have been documented to experience venous migration through intraluminal expansion of the tumor mass which is an unusual extension that follows the vena cava and involves the wall. Tumor thrombus (TT) with RCC has not been demonstrated to be a predictor of survival after surgical intervention, despite its extension. But its chances of survival are low if left untreated [3]. Excision of RCC with extend into the IVC and right cardiac chamber require technical operative challenge. Careful preoperative surgical planning and a multidisciplinary team is required. We present the case report of RCC which extends into the IVC and right atrium (RA) and operative excision of the tumor. This case report adheres to the SCARE 2025 guidelines for reporting surgical case reports [4].

## 2 Case presentation

A 40-years old male presented with shortness of breath during activities for 3 months prior. He was also feeling weak. The patient presented to hospital with severe anemia (hemoglobin 6 g/dL) secondary to anemia of chronic disease related to malignancy. He was admitted and received packed red cell transfusion, resulting in an increase in hemoglobin to 10.5 g/dL. He underwent PET CT Abdominal scan for further evaluation which later showed right kidney tumor and its extension into the heart and consulted to cardiologist for echocardiography. No history of weight loss or haematuria. Past medical history was hypertension. There was no history of diabetes, liver disease or previous kidney disease.

**Fig. 1:**
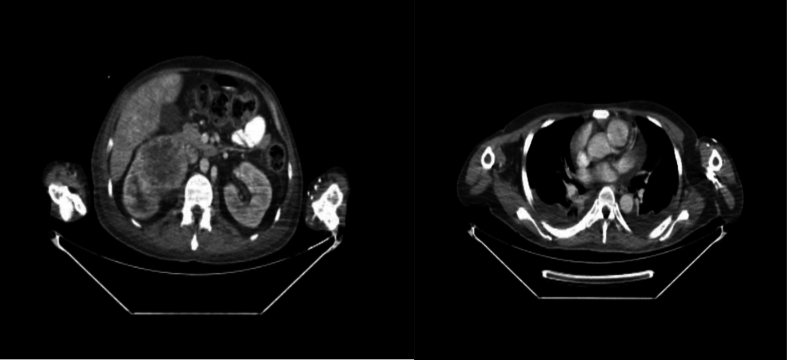
A lobulated hypermetabolic mass with a malignant aspect measuring ±68.6 × 64.9 mm accompanied by a necrotic area in it in the right renal lower pole corresponds to renal carcinoma in the form of RCC. The mass was pressing on the proximal right ureter, causing grade 3 right renal hydronephrosis and pressing on the inferior right renal pelvic. TT of the inferior and superior vena cava that reaches the right atrium is accompanied by widening of the vena cava.

**Fig. 2:**
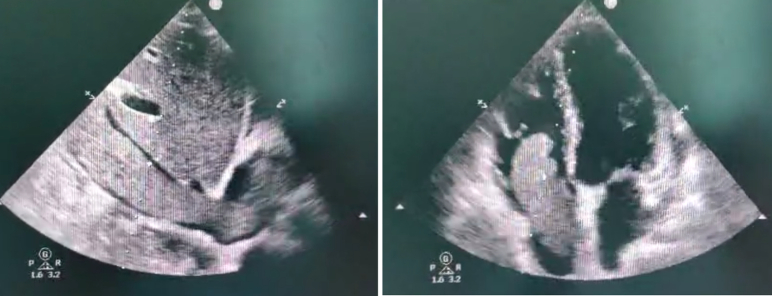
Multiple lobe thrombus from the IVC entering the RA. with the mobile end going in and out of the RV, partial obstruction of the TV (RV inflow) without any evidence of tricuspid valve adherence or invasion.

Initial physical examination showed stable vital sign and did not reveal any abnormal finding. ECG and Chest X-ray showed normal result. Baseline serum creatinine was 0.8 mg/dL, estimated GFR 102 mL/min/1.73 m^2^. NYHA functional class II dyspnea was noted. LVEF was 63 % on echocardiography. PET CT-scan showed A lobulated hypermetabolic mass with a malignant aspect measuring ±68.6 × 64.9 mm accompanied by a necrotic area in it in the right renal lower pole corresponds to renal carcinoma in the form of RCC. The mass was pressing on the proximal right ureter, causing grade 3 right renal hydronephrosis and pressing on the inferior right renal pelvic. TT of the inferior and superior vena cava that reaches the right atrium is accompanied by widening of the vena cava (Fig. 1). Transthoracic echocardiography confirmed a multilobulated, mobile thrombus prolapsing into the RA, partially obstructing right ventricular inflow, without any evidence of tricuspid valve adherence or invasion (Fig. 2).

**Fig. 3:**
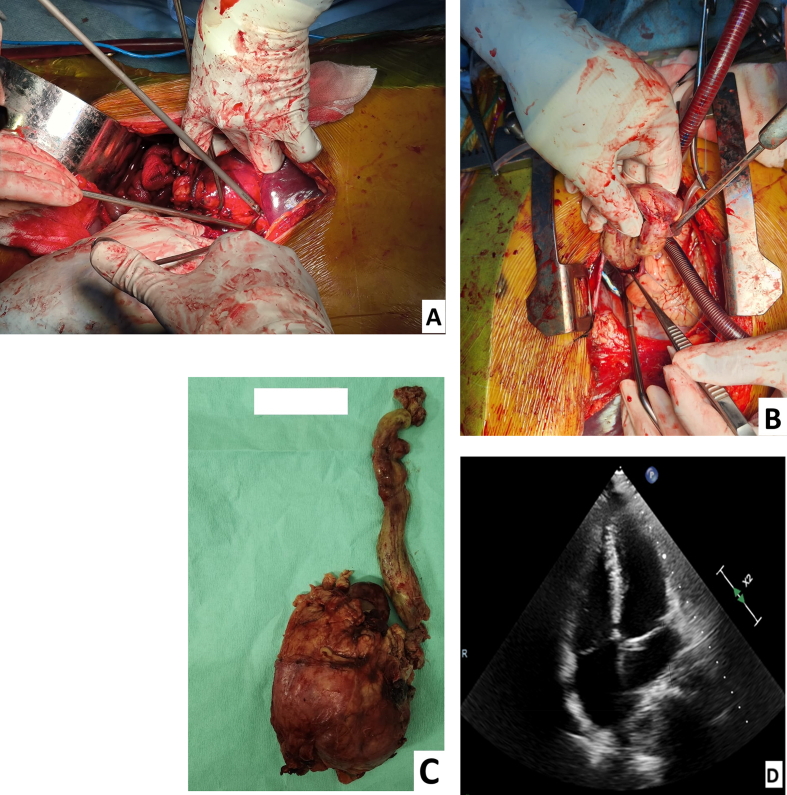
A. The right kidney excision was done. B. Tumor thrombus from IVC was pushed and evacuated through right atrium. C. Right kidney tumor and tumor thrombus extending from IVC to RA. D. TEE showed no sign of tumor thrombus in RA.

Surgical therapy was recommended with the excision of right kidney tumor and TT into the IVC and RA was performed using cardiopulmonary bypass (CPB). CPB was initiated with perfusion flow 2.4 L/min/m^2^ (normothermia 35 °C) because due to ongoing intraoperative bleeding from the nephrectomy bed, continued blood loss, and hemodynamic instability during tumor resection. Aorta was cannulated for inflow, right femoral vein and SVC was cannulated for outflow. After complete resection of right kidney tumor, renal artery was clipped. The TT in the IVC was unable to be evacuated. The IVC and RA were opened, after which the thrombus was mobilised and gently advanced into the right atrium, enabling complete removal under direct vision. The right kidney tumor measured approximately 10.0 × 13.0 cm, with TT was approximately 9.5 cm in length, mobile, without macroscopic caval wall invasion on intraoperative inspection. Intraoperative transoesophageal echocardiography (TEE) guided the extraction, confirming thrombus stability before manipulation and complete clearance thereafter. (Fig. 3). Then IVC was repaired with direct continuous suture. CPB was weaning off until stopped. CPB duration was 74 min. Transoesophageal echocardiography (TEE) showed no residual of TT. The surgery was completed in the usual manner. Estimated blood loss 1200 mL; transfused 3 units PRC, 2 units FFP, 3 units platelets intraoperatively.

Patient the admitted to the cardiac intensive care unit. Post-operatively, dopamine was initiated at 10 μg/kg/min and vasoactive support with norepinephrine 0.06 μg/kg/min and weaned off completely at 40 h. Hemoglobin level was 10.2 g/dL and lactate of 1.8 mmol/L. Patient was transferred from the ICU to the General ward after at 44 h post-operative. He was discharged of post-operative day 6. Histopathology confirmed clear cell RCC and TT through IVC and RA. The patient was commenced on pazopanib 800 mg orally once daily as adjuvant targeted therapy. 3 months later CT-scan with contrast follow up showed there was no radiologic evidence of residual lesion in the nephrectomy bed or along the IVC/RA pathway, and no evidence of metastatic disease.

## 3 Discussion

Renal cell carcinoma was 7.8 % among the secondary cardiac metastasis tumors which can spread through blood vessel route through the inferior vena cava. The incidence was among 4–25 % patient. The symptoms on the patient include sign of cardiac failure due to obstruction of IVC and partial obstruction of the RV inflow [5,6]. Renal cell carcinoma classification has been classified by Robson et.al (Table 1) with stage IIIa with Renal vein and IVC involvement in this case [7]. TNM staging was T3N0M0 with Stage III RCC based on EAU Guidelines on Renal Cell Carcinoma 2024 [8]. Mayo Clinic also described five levels of RCC according to the involvement of renal vein, IVC dan RA for planning the management strategy (Table 2) [9].

**Table 1:** Classification of renal cell carcinoma by Robson et al. [7].

Stage		
I		Confined by renal capsule
II		Extension to perirenal fat but confined to Gerotta's fascia
III	IIIa	Renal vein or IVC involvement
	IIIb	Lymphatic involvement
	IIIc	Combination of IIIa and IIIb
IV	IVa	Spread to contiguous organs except ipsilateral adrenal
	IVb	Distal metastases

**Table 2:** Mayo Clinic RCC tumor thrombus level classification system and potential surgical approach [9].

Level	Description	Surgical approach
0	Limited to renal vein or its tributaries	Renal vein ligation
1	Extends into IVC but <2 cm above renal vein orifice	Milking of IVC tumor thrombus into renal vein followed by renal vein ligation
2	Extends into IVC, >2 cm above renal vein orifice but below hepatic veins	Some mobilization of liver (ligation of accessory hepatic veins draining caudate lobe), clamping of intrahepatic IVC, clamping of intrahepatic IVC and contralateral renal Vein
3	Extends above hepatic veins but below diaphragm	Extensive mobilization of liver, including ligation of diaphragmatic attachments, clamping of suprahepatic IVC with adjunctive venovenous or CPB
4	Extends above diaphragm	Involvement of cardiothoracic surgery, potential thoracotomy and open heart surgery

In level 4 tumor thrombus level, involvement of cardiothoracic surgery and open-heart surgery is required. Imaging is important to determine the best surgical approach of the patient. Several imaging techniques have been proposed. CT scan has high specificity for capsular invasion, nodal disease, and renal vein and IVC TT but has poor sensitivity for capsular invasion and lymph node involvement by tumor. Although MRI is generally more accurate than CT for differentiating tumor thrombus from bland thrombus [10], it was not performed in this case due to the patient's financial limitation, which restricted access to advanced cross-sectional imaging at the time of presentation.

Surgical strategy regarding the level 4 TT can be accomplished with CPB. Whether using DCHA (deep hypothermic circulatory arrest) or not. While DHCA can provide an ideal, bloodless operative field and facilitate prolonged intracardiac or caval work, it is associated with longer operative times, hypothermia-related coagulopathy or platelet dysfunction, and greater human resource demands. Ishiyama et al. compared DHCA and non-DHCA approaches for RCC-related thrombi and found both feasible and safe when appropriately selected. DHCA remains an option when prolonged RA or IVC interventions are anticipated for example, in cases of suspected adhesion to the caval wall, large or friable thrombi, or extension into/around the tricuspid valve [[11], [12], [13]]. In our case, the thrombus morphology short, mobile, non-adherent, confined to the lower right atrium, and without valvular engagement corresponded to lower-risk profiles in which DHCA can be omitted. Preoperative CT confirmed no adhesion to the caval wall. After establishing CPB, we performed a right atriotomy without placing a clamp on the IVC. Under direct vision, the thrombus was gently mobilised and pushed down from the right atrium for complete extraction. Intraoperative transoesophageal echocardiography (TEE) was used selectively before thrombus manipulation and after resection to ensure stability during removal and to confirm the absence of residual tumor. This method aligns with contemporary series emphasising that targeted TEE monitoring supports safe thrombectomy when DHCA is avoided [[11], [12], [13], [14], [15]].

The sole treatment option for people with locally advanced RCC is a highly aggressive surgical technique. When RCC extends to the IVC, surgical management becomes difficult. Surgical management is challenging, particularly in Level IV thrombus, which often requires a multidisciplinary approach. Reported 5-year survival rates for non-metastatic RCC with IVC tumor thrombus range from 34 % to 72 % [9,10,16]. In the series by Vergho et al. [16], patients without metastases achieved a 50.7 % 5-year cancer-specific survival rate. Multiple studies have shown that tumor grade presence of distant metastases and lymph node invasion are independent prognostic factors, whereas thrombus level alone does not significantly influence survival. The absence of metastatic disease in the present case therefore represents a favourable prognostic feature and supports an aggressive surgical approach with curative intent. Furthermore, this status provides a theoretical basis for considering postoperative adjuvant systemic therapy in high-risk, non-metastatic RCC [5,6,[9], [10], [11],^16^]. Subgroups of Level IV patients requiring CPB including those with supradiaphragmatic thrombus have demonstrated 5-year cancer-specific survival rates comparable to other non-metastatic IVC-TT cohorts when metastases are absent [[11], [12], [13], [14], [15], [16]]. These outcomes highlight the oncological safety of aggressive surgical resection with CPB in appropriately selected patients. Our patient's tumor was T3N0M0 clear cell RCC, with no nodal or distant spread a favourable prognostic factor but still classified as high-risk under both EAU 2024 and NCCN 2025 guidelines [8,17]. For such patients, adjuvant systemic therapy is recommended. Patient was commenced on pazopanib 800 mg orally once daily as targeted therapy. This decision reflects a patient-centred approach balancing efficacy, accessibility, and tolerability, and aligns with evidence supporting VEGF-targeted agents in the postoperative setting.

## 4 Conclusion

Surgical management of RCC with tumor thrombus with extension needed to be assessed accurately regarding the tumor level, degree of obstruction, and present of any distant metastases for selected treatment. In supradiaphragmatic cases, early surgical intervention by a coordinated urology–cardiac surgery team, often utilising cardiopulmonary bypass, can provide favourable oncological outcomes in metastasis-free patients. Tailoring the surgical approach to thrombus morphology and caval wall involvement may allow omission of deep hypothermic circulatory arrest, reducing perioperative morbidity without compromising tumor clearance. For high-risk clear-cell RCC, current guidelines recommend considering adjuvant systemic therapy following complete resection to mitigate recurrence risk.

## Author contribution

All of the authors equally contributed to the study from the conceptual framework, data gathering, and data analysis until interpreting the study results.

## Patient consent

Written informed consent was obtained from the patient/legal guardian for publication and any accompanying images. A copy of the written consent is available for review by the Editor-in-Chief of this journal on request.

## Ethical approval

Written informed consent was obtained from the patient for publication and any accompanying images and exempt from ethical approval when the patient provides consent or a guarantee. A copy of the consent is available upon request.

## Guarantor

Muhammad Arza Putra.

## Research registration number

Not applicable.

## Declaration of Generative AI and AI-assisted technologies in the writing process

Language model AI was employed to improve the grammar and spelling checking during manuscript writing.

## Funding

Not applicable.

## Conflict of interest statement

The authors declare that there is no competing interest regarding the manuscript.
